# Michael Kohlhaas Syndrome – Taking the court to court

**DOI:** 10.1192/bjo.2021.353

**Published:** 2021-06-18

**Authors:** Louisa Ward

**Affiliations:** Worcestershire Health and Care NHS trust

## Abstract

**Objective:**

To explore Michael Kohlhaas syndrome and development of litigious paranoia.

**Case report:**

A 50-year-old Swiss woman spent a number of weeks travelling around the UK, reporting to police that her family in Switzerland were working with their police, doctors and the mafia to kill her. She was therefore running away for her own safety, and trying to seek legal help to investigate. She had been in close contact with a woman in London who claimed to be able to give her legal aid in exchange for payment. On admission to the ward, it was felt that the legal aid was actually feeding her persecutory delusions.

**Discussion:**

We discovered that she had in fact been detained in Switzerland prior to coming to the UK, and the discharge report was obtained which diagnosed her with Michael Kohlhaas syndrome and Folie a deux.

**Conclusion:**

This poster will further explore Michael Kohlhaas syndrome and litigious paranoia, with connection to this case.

